# Supplementary material for: Content-rich biological network constructed by mining PubMed abstracts
Source: BMC Bioinformatics. 2004 Oct 8;5:147. doi: 10.1186/1471-2105-5-147 (PMC528731; doi:10.1186/1471-2105-5-147)
Supplement: Additional File 5 — The original Chilibot query results of the term "long-term potentiation (LTP)" and 22 other terms, limiting the latest references analyzed to the years 1990, 1995, 2000, and 2004. [file 1471-2105-5-147-S5.bz2 › chilibotAdditionalFile5/ltp2004/html/LTP_SYNAPSIN I.html]

 


 **LTP** and **SYNAPSIN I** 
  
Found 21 abstracts in PubMed,  **21 abstracts were retrieved and analyzed**.  


---

 Search Google  |
 PDF files only 
|  EDU domain only 

---

**Interactive relationship** (e.g. stimulation, inhibition, etc)

**Parallel relationship** (e.g. studied together, co-existance, homology, etc.)

- Our results indicate that  **LTP**  expression is accompanied by persistent changes in presynaptic phosphorylation, and specifically that presynaptic CaM kinase II activity and  **synapsin I**  phosphorylation may be involved in  **LTP**  expression.  Ref: 8986832 Proc Natl Acad Sci U S A, 1996
- We studied the time dependent changes in  **synapsin I**  mRNA levels after hippocampal long term potentiation  [ **LTP** ]   **LTP**  in rats in vivo.  Ref: 9479047 Brain Res, 1998
- Immunocytochemical staining revealed that protein levels for both  **synapsin I**  and syntaxin 1B were elevated in the mossy fibre terminal zone of CA3 5 h after the induction of long term potentiation  [ **LTP** ] .  Ref: 9200718 Neuroscience, 1997
- The level of  **synapsin I** , a synaptic vesicle protein and marker for synaptic activation, was studied 8 h after long term potentiation  [ **LTP** ]   **LTP**  induction in rat hippocampus in vivo.  Ref: 10924697 Brain Res, 2000
- These results suggest that glutamate can activate CaM kinase II through NMDA receptors in the induction of  **LTP**  and in turn stimulates the phosphorylation of target proteins such as MAP2 and  **synapsin I** .  Ref: 8282267 Nippon Yakurigaku Zasshi, 1993
- calcium calmodulin dependent protein kinase II phosphorylation of the presynaptic protein  **synapsin I**  is persistently increased during long term potentiation  [ **LTP** ] .  Ref: 8986832 Proc Natl Acad Sci U S A, 1996
- These results suggest that the increased level of  **synapsin I**  mRNA is related to persistent enhancement of synaptic activity within the neural networks in which dentate granule cells participate in  **LTP** .  Ref: 9479047 Brain Res, 1998
- We utilized a novel multiple electrode stimulator to produce  **LTP**  in a substantial portion of the synapses in a hippocampal CA1 minislice and tested the effects of such stimulation on the presynaptic protein  **synapsin I** .  Ref: 8986832 Proc Natl Acad Sci U S A, 1996
- Following  **LTP**  induction by stimulating the perforant path,  **synapsin I**  mRNA expression in the granule cell layer of the dentate gyrus ipsilateral to stimulation increased significantly in a time dependent manner.  Ref: 9479047 Brain Res, 1998
- Increased  **synapsin I**  immunoreactivity during long term potentiation  [ **LTP** ]  in rat hippocampus.  Ref: 10924697 Brain Res, 2000
- These results support the idea that CaM kinase II contributes to the induction of hippocampal  **LTP**  in both postsynaptic and presynaptic regions through phosphorylation of target proteins such as the AMPA receptor, MAP2 and  **synapsin I** .  Ref: 8740440 Neurochem Int, 1996
- Time dependent changes in rat hippocampal  **synapsin I**  mRNA expression during long term potentiation  [ **LTP** ] .  Ref: 9479047 Brain Res, 1998
- Electrophysiology reveals that mice lacking  **synapsin I**  exhibit a selective increase in paired pulse facilitation, with no major alterations in other synaptic parameters such as long term potentiation  [ **LTP** ] .  Ref: 7902212 Cell, 1993
- The levels of messenger RNA encoding both  **synapsin I**  and syntaxin 1B were increased on the ipsilateral side of the dorsal dentate gyrus 2 and 5 h following the induction of long term potentiation  [ **LTP** ] .  Ref: 9200718 Neuroscience, 1997
- **Synapsin I**  and syntaxin 1B key elements in the control of neurotransmitter release are regulated by neuronal activation and long term potentiation  [ **LTP** ]  in vivo.  Ref: 9200718 Neuroscience, 1997
- Studies of knockout mice have shown that the synaptic vesicle protein Rab3A is required for mossy fibre  **LTP** , but the protein kinase A substrates rabphilin,  **synapsin I**  and synapsin II are dispensable.  Ref: 11797010 Nature, 2002
- We have previously shown that the induction of long term potentiation  [ **LTP** ]   **LTP**  at perforant path to dentate granule cell synapses in the rat hippocampus induces changes in the mRNA levels of syntaxin 1B and  **synapsin I** , known to be involved in neurotransmitter release.  Ref: 9749751 Eur J Neurosci, 1998
- Regulation of neuronal plasticity by phosphorylation of  **synapsin I**  and of postsynaptic substrates necessary for long term potentiation  [ **LTP** ]  is another dynamic area of investigation.  Ref: 1323238 Annu Rev Biochem, 1992
- **LTP**  inducing stimulation produced a long lasting 6 fold increase in the phosphorylation of  **synapsin I**  at its calcium calmodulin dependent protein kinase II CaM kinase II sites without affecting  **synapsin I**  levels.  Ref: 8986832 Proc Natl Acad Sci U S A, 1996
